# Biomonitoring Data for 2,4-Dichlorophenoxyacetic Acid in the United States and Canada: Interpretation in a Public Health Risk Assessment Context Using Biomonitoring Equivalents

**DOI:** 10.1289/ehp.0900970

**Published:** 2009-08-12

**Authors:** Lesa L. Aylward, Marsha K. Morgan, Tye E. Arbuckle, Dana B. Barr, Carol J. Burns, Bruce H. Alexander, Sean M. Hays

**Affiliations:** 1 Summit Toxicology, LLP, Falls Church, Virginia, USA; 2 U.S. Environmental Protection Agency, Research Triangle Park, North Carolina, USA; 3 Environmental Health Science and Research Bureau, Health Canada, Ottawa, Ontario, Canada; 4 National Center for Environmental Health, Centers for Disease Control and Prevention, Atlanta, Georgia, USA; 5 Dow Chemical Company, Midland, Michigan, USA; 6 Division of Environmental Health Sciences, School of Public Health, University of Minnesota, Minneapolis, Minnesota, USA; 7 Summit Toxicology, LLP, Lyons, Colorado, USA

**Keywords:** 2,4-dichlorophenoxyacetic acid, biomonitoring, exposure biomarkers, exposure monitoring, risk assessment

## Abstract

**Background:**

Several extensive studies of exposure to 2,4-dichlorophenoxyacetic acid (2,4-D) using urinary concentrations in samples from the general population, farm applicators, and farm family members are now available. Reference doses (RfDs) exist for 2,4-D, and Biomonitoring Equivalents (BEs; concentrations in urine or plasma that are consistent with those RfDs) for 2,4-D have recently been derived and published.

**Objective:**

We reviewed the available biomonitoring data for 2,4-D from the United States and Canada and compared them with BE values to draw conclusions regarding the margin of safety for 2,4-D exposures within each population group.

**Data sources:**

Data on urinary 2,4-D excretion in general and target populations from recent published studies are tabulated and the derivation of BE values for 2,4-D summarized.

**Data synthesis:**

The biomonitoring data indicate margins of safety (ratio of BE value to biomarker concentration) of approximately 200 at the central tendency and 50 at the extremes in the general population. Median exposures for applicators and their family members during periods of use appear to be well within acute exposure guidance values.

**Conclusions:**

Biomonitoring data from these studies indicate that current exposures to 2,4-D are below applicable exposure guidance values. This review demonstrates the value of biomonitoring data in assessing population exposures in the context of existing risk assessments using the BE approach. Risk managers can use this approach to integrate the available biomonitoring data into an overall assessment of current risk management practices for 2,4-D.

Biomonitoring data for 2,4-dichlorophenoxyacetic acid (2,4-D) in urine samples are now available from a number of studies of both the general population (including preschool-age children) and farm applicators and their family members [Alexander BH, et al. 2007; Arbuckle and Ritter 2005; Arbuckle et al. 2002, 2004, 2006; Centers for Disease Control and Prevention (CDC) 2005; Morgan et al. 2008]. Such data provide an integrated measure of absorbed dose from all pathways and routes of exposure. The hazards of 2,4-D were recently assessed by the U.S. Environmental Protection Agency (U.S. EPA 2004) and the Canadian Pest Management Regulatory Agency (PMRA 2007). The U.S. EPA–derived reference doses (RfDs) for acute and chronic exposure to 2,4-D are based on external exposure metrics (administered dose), which are not directly useful for evaluating biomonitoring data. However, Biomonitoring Equivalent (BE) values corresponding to RfDs for acute and chronic exposure scenarios are now available (Aylward and Hays 2008) and can be used as a tool for assessing the biomonitoring data directly in a public health risk assessment context, without requiring calculation of corresponding external dose, as has previously been done (Mage et al. 2004). Here we review urinary biomonitoring data for 2,4-D from several studies in the general population and in farmers and farm family members and evaluates the data in the context of the BE values for 2,4-D presented by Aylward and Hays (2008) to assess the current margin of safety (ratio of exposure guidance value such as an RfD to exposure measures) for population exposures to 2,4-D in the United States and Canada.

## Methods

### Biomonitoring data

We used urinary biomonitoring data for 2,4-D from several studies of both general population adults and children and from studies of farmers and farm family members, as follows.

The National Center for Environmental Health of the Centers for Disease Control and Prevention (CDC 2005) measured 2,4-D in urine samples collected from a complex, stratified random sample of the civilian, noninstitutionalized population of the United States, 6–59 years of age, during 2001–2002, as part of the National Health and Nutrition Examination Survey (NHANES).

Morgan et al. (2004, 2008) recently examined the exposures of 135 preschool children and their adult caregivers to 2,4-D at their homes in North Carolina and Ohio from the Children’s Total Exposure to Persistent Pesticides and Other Persistent Organic Pollutants (CTEPP) study. Participants were randomly recruited from homes in six North Carolina and six Ohio counties. Participants were recruited by field staff from homes between February 2000 and February 2001 in North Carolina and January 2001 and November 2001 in Ohio. Monitoring was performed over a 48-hr period at the participants’ homes. Spot urine samples and environmental samples including air, soil, dust, hand wipes, and food were collected and analyzed for 2,4-D.

Alexander BH, et al. (2007) reported urinary 2,4-D data from the Farm Family Exposure Study. Participants in the study included 34 farmers in Minnesota and South Carolina who were licensed applicators and their spouses and children (*n* = 53) living on the farm property. Participants collected 24-hr urine samples the day before, the day of, and for 3 days after application of 2,4-D on their farms during the 2000 or 2001 growing season.

Curwin et al. (2005) measured urinary 2,4-D concentrations in 16 farmers 1–5 days after their application of 2,4-D on the farm during the spring and summer of 2001. The evening and the following first morning urine samples were composited.

The Pesticide Exposure Assessment Study measured the extent to which agricultural pesticide applicators and their families in Ontario, Canada, are exposed to pesticides during normal handling practices (Arbuckle and Ritter 2005; Arbuckle et al. 2002, 2004). Farmers from the previously conducted Ontario Farm Family Health Study (Arbuckle et al. 1999) that had reported using phenoxyacetic acid herbicides were telephoned in early 1996 to determine their eligibility for the Pesticide Exposure Assessment Study. To be eligible, the farmer had to *a*) be planning to use 2,4-D or (4-chloro- 2-methylphenoxy)acetic acid (MCPA) in the coming growing season, *b*) be the individual who would be handling the herbicides on the farm, *c*) have his or her home on the farm property, and *d*) be currently living with his or her spouse. A total of 126 families provided a spot urine sample before handling either 2,4-D or MCPA and then provided two consecutive 24-hr samples after use of the herbicide. All samples were collected in 1996.

The Agricultural Health Study (AHS)/Pesticide Exposure Study (PES) was designed to evaluate exposure to 2,4-D and chlorpyrifos in a subset of individuals enrolled in the AHS, which is a large, prospective epidemiologic study of pesticide applicators and their spouses in Iowa and North Carolina designed to assess the relationships between agricultural exposures and disease. Participants in the AHS were contacted randomly and surveyed to ascertain their planned use of the 2,4-D and chlorpyrifos, and then a subset of participants were enrolled in the PES (Thomas et al. 2009). Urinary samples were collected during 2001 and 2002 and included a preapplication first morning void sample, as well as a 24-hr sample starting the day of application (day 1) and, optionally, for days 2–5 as well.

Descriptions of the institutional review board approvals and informed consent information for each of these studies are presented in the cited publications.

### RfDs and biomonitoring equivalents

The U.S. EPA recently conducted a review of 2,4-D and adopted both a chronic oral RfD as well as acute RfDs (applicable to single-day exposures) for this herbicide (U.S. EPA 2004). Table 1 summarizes the derivations of the BE values associated with the RfD values. BEs are defined as the concentration of a chemical or its metabolite in a human biological medium (usually blood or urine) that is consistent with existing exposure guidance values. BE values are screening values corresponding to existing risk assessments and not intended for use as definitive measures of risk for individuals. A full description of the BE approach and application is beyond the scope of this review but is presented elsewhere (Hays and Aylward 2009; Hays et al. 2007, 2008).

The pharmacokinetics of 2,4-D have been studied in two sets of human volunteers (Kohli et al. 1974; Sauerhoff et al. 1977). Both studies found that 2,4-D is eliminated in urine either as the unchanged parent compound (80–95%) or as a conjugate, with urinary half-lives on the order of 1 day. There was no evidence of oxidative metabolism, consistent with data from other mammalian species (Timchalk 2004). Based on these pharmacokinetic data, continuing exposure for more than 1 week of exposure would result in a steady state in which the amount excreted daily in urine would be approximately equivalent to the amount absorbed each day.

Because 2,4-D is excreted as the parent compound in urine, most biomonitoring evaluations of exposure to 2,4-D have relied on measurements (quantifying both free and conjugated parent compound) in urine samples (CDC 2005; Knopp 1994; Knopp and Glass 1991), although a few kinetic studies have also examined plasma concentrations of 2,4-D in humans and animals (Kohli et al. 1974; Saghir et al. 2006; Sauerhoff et al. 1977; van Ravenzwaay et al. 2003). The relative ease of collection of urine samples compared with blood samples contributes to this choice. From a toxicologic point of view, plasma concentrations of 2,4-D are probably more informative for predicting target tissue concentrations and responses (e.g., neurotoxic responses). This would be particularly true under conditions of episodic, higher-level exposures. However, under conditions of chronic, low-level exposures, urinary excretion rates of 2,4-D should be specific and quantitatively relevant in a framework of a mass-balance assessment. That is, under exposure conditions that approximate steady-state conditions [consistent with the definition of chronic RfDs and related exposure guidance values; see, e.g., the definition of RfD provided under the U.S. EPA Integrated Risk Information System program (U.S. EPA 2009)], daily urinary excretion of 2,4-D should equal daily intake.

The straightforward elimination kinetics of 2,4-D (as parent compound or conjugate in urine with essentially no oxidative metabolism) and the lack of direct relationship between urinary concentration and critical internal dose metrics suggest a simple mass-balance approach for derivation of BE values for urinary 2,4-D consistent with chronic exposure at the chronic RfD. The process of deriving the BE_POD_ and BE_RfD_ values for 2,4-D is detailed by Aylward and Hays (2008) and summarized below and in Table 1.

The point of departure (POD) for the U.S. EPA chronic RfD is a no observed adverse effect level (NOAEL) of 5 mg/kg-day in rats fed 2,4-D chronically in the diet. Applying an uncertainty factor (UF) of 10 for interspecies variation, the human equivalent POD is 0.5 mg/kg-day. Calculating the average concentration of 2,4-D in urine in humans associated with this chronic daily dose (after application of the interspecies UF) yields the BE_POD_. The daily mass intake at the human equivalent POD was estimated for a variety of child and adult body weights. Estimated distributions of daily creatinine excretion or urinary volume as a function of sex, age, and body size were used in a Monte Carlo analysis to estimate a distribution of creatinine-adjusted urinary 2,4-D concentrations for various age and sex categories [methods are described in detail by Aylward and Hays (2008)]. The average of median estimated creatinine-adjusted 2,4-D concentration consistent with chronic exposure at the human-equivalent POD (BE_POD_) for 2,4-D for adults (males and females) is approximately 20,000 μg/L or 30,000 μg/g creatinine. These values were consistent with the range of median values identified in the simulations for children of various ages. Concentrations at the 95th percentiles of the estimated distributions were generally within a factor of 2 of the median values.

The BE associated with the chronic RfD was derived by dividing the BE_POD_, which reflects the interspecies UF of 10, by the UF of 10 for intraspecies variation and the UF of 10 applied by U.S. EPA for database uncertainties (for a total composite UF from the animal POD of 1,000 applied to the animal NOAEL POD). BE values corresponding to the acute RfDs were derived in a similar fashion, except that steady state was not assumed. Based on the urinary elimination half-life of approximately 1 day, an assumption was made that one-half of the intake dose at the human equivalent POD for the acute RfD values would be eliminated in the first 24 hr after exposures. Average urinary 2,4-D concentrations (both absolute and creatinine adjusted) corresponding to one-half the human equivalent POD doses were estimated, and the intraspecies and database UFs were then applied to obtain the BE_RfD_acute_ values. These BE values are appropriate for use when the exposure is short term and episodic and the timing of the sample collection compared with exposure is known. Table 1 summarizes the derivation and resulting values.

## Results

**Figure 1:**
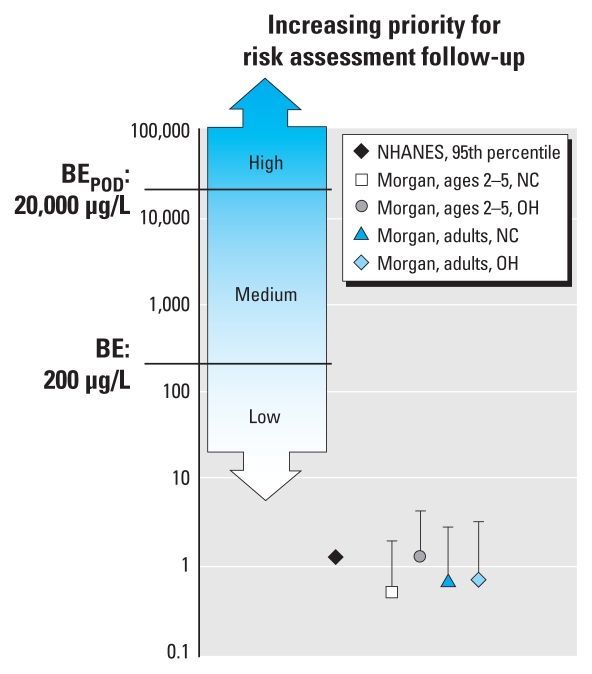
Urinary 2,4-D concentrations (μg/L) in general population studies presented in the context of the BE value corresponding to the U.S. EPA RfD for general population chronic exposures. The symbol for data from NHANES (CDC 2005) represents the 95th percentile for all tested participants (median values were below the LOD; see Table 2). The symbols for data from Morgan et al. (2008) (in key, Morgan) represent the median values for the children and adults from two states; bars extend to the 95th percentile for each group. The shaded regions represent concentration ranges associated with low, medium, and high priority for risk assessment follow-up based on the criteria described in the BE communications guidelines (LaKind et al. 2008).

Table 2 summarizes urinary 2,4-D concentrations measured in studies of general population groups (CDC 2005; Morgan et al. 2008). Exposure pathways for persons in the general population may include ingestion of residues in food products, inhalation, and direct contact with dust (Morgan et al. 2004, 2008). Figure 1 presents the measured urinary concentrations in the context of the appropriate BE values based on the U.S. EPA chronic RfD. The urinary levels of 2,4-D observed in the general population samples are far below the BE value corresponding to the U.S. EPA chronic RfD, with median and upper bound measured concentrations more than 100- and 50-fold below the BE_RfD_.

Table 3 summarizes the corresponding data for farmers and members of their families obtained in the days immediately after application of 2,4-D. Exposure pathways for nonapplicators on the farm may include secondary exposure to treated fields, farm machinery, or the applicator, and drift of herbicide during application with resulting inhalation, dermal, and oral exposure after contact with residues on surfaces in the home. Urinary concentrations collected from farm family members in the day or days immediately after application of 2,4-D fell below the applicable acute BE values.

**Figure 2:**
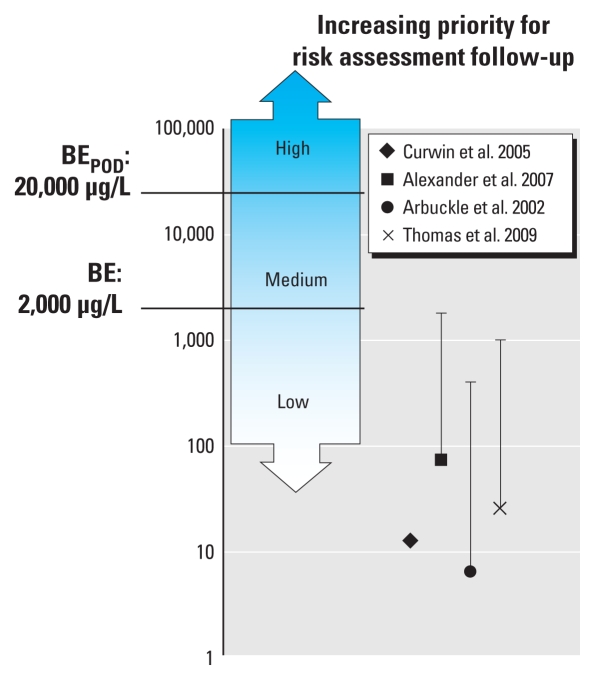
Urinary 2,4-D concentrations (μg/L) in applicators on the day after application of 2,4-D presented in the context of the human-equivalent BE_POD_ and target BE values associated with the occupational risk assessment (U.S. EPA 2004) (see Table 1). Symbols represent the median (or, in the case of Curwin et al. 2005 and Thomas et al. 2009, the geometric mean), and the bars extend to the maximum measured value in each study (not reported for Curwin et al. 2005). For description of shaded regions, see Figure 1 legend.

Figure 2 presents measured urinary concentrations in farmers involved in application of 2,4-D in the context of BE values corresponding to the U.S. EPA occupational exposure guidance values. Again, the data suggest an overall margin of safety, with median or geometric mean levels in farmers involved in application of 2,4-D more than 25-fold below the occupational BE target value. However, some individuals had single spot urinary concentrations that approached the occupational BE target value. The highest urinary level of 2,4-D reported in Thomas et al. (2009) on days 1–5 after application was 2,500 μg/L, in excess of the occupational BE value of 2,000 μg/L (data not shown). However, all other reported occupational measurements were below the occupational BE.

## Discussion

Available biomonitoring data for 2,4-D in both the general and agricultural populations indicate that current uses and practices suggest exposures that are below the acceptable exposures identified by the U.S. EPA. A “margin of safety” is the ratio between the exposure guidance value and measured exposure. In this analysis, the exposure guidance value (RfD) was converted to a BE_RfD_ value for comparison with the measured biomarker concentrations. General population values indicate a margin of safety compared with the BE_RfD_ of approximately 200 at the central tendency and > 50 at the upper percentiles of exposure. In turn, the BE_RfD_ is 100-fold below the BE_POD_, which is the biomarker concentration associated with chronic intake in humans at the POD extrapolated from animals to humans. The conclusion of a substantial margin of safety holds whether comparisons are made using volume or creatinine-adjusted concentrations. Median or average urinary 2,4-D concentrations for applicators are consistently below the BE values associated with occupational exposure targets set by the U.S. EPA (2004); however, evidence exists for exceptions near the occupational BE target value in a few individuals from the studied occupationally exposed populations. Biomonitoring data for spouses and children of applicators on the day after use of 2,4-D also are less than the BE values associated with general population acute exposure RfDs set by the U.S. EPA (2004).

Other studies have reported related biomonitoring data. Arcury et al. (2007) studied children from North Carolina farm worker families in 2004. Multiple pesticides (or metabolites) were measured in urine samples from these children (1–6 years of age). The median 2,4-D concentration was below the limit of detection (LOD) of 0.2 μg/L (42% of the 60 sampled children had detectable concentrations of 2,4-D, but the range of detected concentrations was not reported). Garry et al. (2001) measured urinary 2,4-D in small numbers of forestry applicators who used a variety of methods to apply the herbicide. Backpack sprayers had the highest measured urinary concentrations during time periods of use, with a median of 160 μg/L and a range up to 1,700 μg/L (*n* = 7). Other modes of application such as use of boom sprayers or aerial applications resulted in lower urinary 2,4-D concentrations, with all measured values < 500 μg/L for boom sprayers and < 100 μg/L for other modes. These values are consistent with the concentrations observed in farm applicators from the Alexander BH, et al. (2007) study and are also below the occupational BE_RfD_ presented in Table 1.

The evaluation presented here is based on BE values derived from the U.S. EPA risk assessment of 2,4-D (U.S. EPA 2004). However, the Canadian PMRA has also recently estimated acceptable daily exposures to 2,4-D (PMRA 2007). The derived acute and chronic RfDs are based on the same underlying data as used by the U.S. EPA, with similar or identical choices of POD. However, the PMRA assessment generally applied total UFs approximately 3-fold lower than those applied by the U.S. EPA, resulting in exposure estimates that are approximately 3-fold greater than those set by the U.S. EPA. Thus, the BE_POD_ values associated with the PMRA risk assessment would be essentially identical to those for the corresponding U.S. EPA exposure guidance values. Although BE values were not specifically derived based on the PMRA assessments, corresponding urinary BE values would be approximately 3-fold higher than those derived based on the U.S. EPA RfDs. BE values corresponding to the PMRA acute RfD values for acute exposure in the general population and in females of reproductive age equal to 1,000 and 4,000 μg/L, respectively (2,000 and 7,000 μg/g creatinine). The BE value corresponding to the PMRA acceptable daily intake for chronic exposure would be 700 μg/L (1,000 μg/g creatinine). Thus, reliance on the PMRA risk assessment does not change the overall conclusion of a substantial margin of safety under the various exposure scenarios.

### Uncertainties and limitations

BE values are derived based on expected average concentrations (either volume based or creatinine adjusted) in urine under conditions consistent with the underlying exposure guidance value (chronic or acute exposure conditions). Some variability in concentration is expected because of use of spot urine samples, interindividual variability in creatinine excretion rates, and variability in urinary volume due to hydration status. Morgan et al. (2004, 2008) investigated the variability of 2,4-D concentrations among spot urine samples (i.e., first morning void, after lunch, and before bedtime) collected over the course of 48 hr from 28 adults and 28 children. The maximum measured spot urine value was within a factor of 3 of the mean value in 53 of the 56 individuals, consistent with previous assessments of variability among spot samples (e.g., Scher et al. 2007).

2,4-D is relatively short-lived, with a urinary half-life on the order of 1 day, so for an individual in the general population, a single measurement does not characterize long-term exposure. However, the NHANES urinary data for 2,4-D are representative of the U.S. population, and samples were collected at various times through the year. NHANES data would be expected to capture indications of higher exposures if they were occurring with any frequency, unless such variations were highly seasonal and geographically isolated. Urinary concentration data from Morgan et al. (2004, 2008) collected from two different geographical regions of the United States (North Carolina and Ohio) over the course of a year suggest somewhat higher exposures than reflected in the NHANES data set, but both sets indicate general population exposures far below health-based exposure guidance values.

A notable deficit in the available data for the general population pertains to residential uses of 2,4-D. Unlike exposures to 2,4-D users in agricultural populations, systematic evaluations of domestic use of the chemical are not available. These episodic exposures would not likely be captured in the NHANES (CDC 2005) or Morgan et al. (2008) data. To the extent that domestic applications do not result in exposures greater than those resulting from agricultural applications, human exposures should be within the margin of safety demonstrated by these existing study data. More research is needed to understand the patterns of domestic use of 2,4-D in residential settings and the resulting potential human exposures to this herbicide in the United States and Canada.

The RfD values derived by the U.S. EPA are based on noncancer end points. 2,4-D has also been assessed for potential carcinogenic effects. Non-Hodgkin lymphoma (NHL) was associated with herbicides and 2,4-D in a series of case–control studies initiated > 20 years ago (Hoar et al. 1986; Zahm et al. 1990). Subsequent case–control and cohort studies have not confirmed these early observations (Burns et al. 2001; De Roos et al. 2003; Hartge et al. 2005; Pearce 1989; Schroeder et al. 2001; Woods et al. 1987). Recent reviews of NHL (Alexander DD, et al. 2007) and 2,4-D (Garabrant and Philbert 2002) have concluded that the epidemiologic evidence remains “scant” and unsupportive for this association.

BE values are screening values and are not intended for use as definitive measures of risk for individuals. They do not represent a bright line between safe and unsafe levels, but rather allow evaluation of biomonitoring data in a public health risk context consistent with the existing risk assessment for 2,4-D (LaKind et al. 2008). Biomarker concentrations below the BE_RfD_ indicate a low priority for risk assessment follow-up, whereas concentrations in excess of the BE_RfD_ but below the BE_POD_ indicate a medium priority for risk assessment follow-up. Values in excess of the BE_POD_ indicate a high priority for risk assessment follow-up. Risk assessment follow-up may include examination of the underlying risk assessment, exposure pathway investigations, or other risk management activities (LaKind et al. 2008). Acute RfDs and the corresponding BE values are targeted at isolated, single-day exposures and are appropriate for use in evaluating biomonitoring data only when there is specific knowledge of a potential acute exposure. The biomonitoring data reviewed here for both members of the general population and applicators generally falls into the range of low priority for risk assessment follow-up, according to the guidelines for BE communication (LaKind et al. 2008).

## Conclusions

Considerable population-level and microlevel data are now available regarding domestic and agricultural exposures to 2,4-D as measured by urinary 2,4-D excretion. These data suggest that current use patterns and risk management efforts by industry and government are likely keeping average exposure to 2,4-D for the general population and in farm family members, and likely other persons potentially exposed from proximity to use of this herbicide, to levels well below current noncancer reference values established both by the U.S. EPA’s Office of Pesticide Programs and by Canada’s PMRA.

## Figures and Tables

**Table 1:** RfDs established by the U.S. EPA (2004) for 2,4-D and derivation of corresponding BE values.

	RfD			
		Acute		
Reference value	Chronic	Females of reproductive age	Other general population	Occupational exposure^a^
Underlying study type	Rat chronic dietary bioassay	Rat oral gavage, gestational days 6–15	Rat acute gavage	Rat chronic dietary bioassay
End point	Decreased body weight gain and food consumption, alterations in hematology and clinical chemistry parameters, increased thyroid weights, and decreased testes and ovarian weights	Skeletal variations and malformations	Gait abnormalities	Same as for chronic RfD
POD (NOAEL) (mg/kg-day)	5	25	67	5
Interspecies UF	10	10	10	10
Human equivalent POD (mg/kg-day)	0.5	2.5	6.7	0.5
BE_POD_ (urinary 2,4-D)	20,000 μg/L (30,000 μg/g cr)	40,000 μg/L (70,000 μg/g cr)	100,000 μg/L (200,000 μg/g cr)	20,000 μg/L (30,000 μg/g cr)
Intraspecies UF	10	10	10	10
Database UF^b^	10	10	10	NA
BE_RfD_ (urinary 2,4-D)	200 μg/L (300 μg/g cr)	400 μg/L (700 μg/g cr)	1,000 μg/L (2.000 μg/g cr)	2,000 μg/L (3,000 μg/g cr)

Abbreviations: cr, creatinine; NA, not applicable; NOAEL, no obseverd adverse effects level; POD, point of departure; UF, uncertainty factor. Details of the derivation are presented by Aylward and Hays (2008).

a Derivation based on U.S. EPA (2004) memorandum indicating *a*) POD same as for general population chronic RfD, and *b*) desired margin of exposure (ratio between POD and exposure level) of 100, based on UFs of 10 each for inter- and intraspecies variation.

b UF applied to account for the lack of a developmental neurotoxicity study and the need for a repeated two-generation bioassay with a focus on thyroid and immunotoxicity end points.

**Table 2:** Urinary biomonitoring data for samples from the general U.S. population.

Study (*n*)	Age group (years), population	Sample description	Percentile			
			μg/L		μg/g cr	
			50th	95th	50th	95th
NHANES, 2001–2002 (CDC 2005)						
546	6–11, USA	Spot	< LOD^a^	1.55	< LOD	1.40
797	12–19, USA	Spot	< LOD	1.24	< LOD	0.662
1,070	20–59, USA	Spot	< LOD	1.27	< LOD	1.04
2,413	All, 6–59, USA	Spot	< LOD	1.27	< LOD	1.08
Morgan et al. (2008)						
66	2–5, NC	48-hr composites	0.5	1.9	1.0^b^	3.4^b^
69	2–5, OH	48-hr composites	1.2	4.3	1.5^c^	5.1^c^
66	20–44, NC	48-hr composites	0.7	2.8	0.6^b^	2.3^b^
69	19–49, OH	48-hr composites	0.7	3.3	0.5^c^	3.3^c^

LOD, limit of detection.

a LOD for NHANES 2001–2002 was 0.2 μg/L.

b *n* = 55.

c *n* = 59.

**Table 3:** Concentrations of 2,4-D measured in urine collected after acute exposure due to agricultural use of 2,4-D.

	Median (range)			
Group, *n*	μg/L	μg/g cr	Sample type	Study
Applicators				
34	73.1 (1.5–1,856)	45.8 (1.1–533.8)	24 hr	Alexander BH, et al. 2007
43	6.0 (0.5–410.0)	NR	24 hr	Arbuckle et al. 2002
16	13^a^ (NR)	NR	Composite of evening and following morning spot samples	Curwin et al. 2005
28	26^b^ (2.2–1,000)	NR	24 hr	Thomas et al. 2009
Spouses^c^				
34	1.2 (0.5–20)	1.1 (0.2–13.1)	24 hr	Alexander BH, et al. 2007
43	< LOD^d^ (< LOD to 61)	NR	24 hr	Arbuckle and Ritter 2005
Children ages 4–17 years				
52	2.9 (0.5–640.4)	2.3 (0.3–660.2)	24 hr	Alexander BH, et al. 2007
Children ages 3–18 years				
91	< LOD^d^ (< LOD to 12)	NR	24 hr	Arbuckle et al. 2004

NR, not reported. Concentrations reported are 2,4-D in urine samples collected 1 day after application of 2,4-D on farms in applicators (Alexander BH, et al. 2007; Arbuckle et al. 2002; Thomas et al. 2009) and family members (spouses and children; Alexander BH, et al. 2007; Arbuckle et al. 2004) or in applicators 1–5 days after application (Curwin et al. 2005).

a Geometric mean for farmers who reported spraying 2,4-D themselves in the previous 1–5 days.

b Geometric mean.

c All spouses were female, and all applicators were male.

d LOD = 1 μg/L.
